# Investigation on the accuracy of field widths and the quantitative relationship with energy variations for dynamic jaws helical tomotherapy

**DOI:** 10.1002/acm2.14251

**Published:** 2023-12-22

**Authors:** Ho Ming Poon, Tin Lok Chiu, Siu Ki Yu

**Affiliations:** ^1^ Medical Physics Department Hong Kong Sanatorium & Hospital Hong Kong SAR China

**Keywords:** dynamic jaws, energy variation, field width, radixact, TomoEDGE, tomotherapy

## Abstract

**Background and purpose:**

TomoEDGE is an advanced technology for TomoTherapy treatment delivery by introducing a sliding‐window dynamic jaw motion. The front and back jaws move independently at the start and end of a target volume along the longitudinal couch direction to reduce the undesired dose to the normal tissues. The accuracy of field width is essential to treatment delivery in this regard. The purpose of this work was to analyze the performance of dynamic jaws on helical tomotherapy and investigate the relationship with energy variation.

**Methods:**

The Tomotherapy‐Quality‐Assurance (TQA) Dynamic Field Width procedure was performed monthly across three tomotherapy machines. All field widths were analyzed, especially the FWHM of the 10 mm field width. Field width measurements were compared with the ratio of Percentage Depth Dose at 20 and 10 cm to render the value of correlation. Changes in beam FWHM and energy were further discussed. Two‐year data were collected for this purpose.

**Results:**

On average, measured field widths in each unit agreed within 1% tolerance recommendation stated. The average absolute difference between reference and measured FWs in each unit was approximately 0.07 mm. An increase of 1.5% in the FW of the 10 mm nominal beam width was correlated with a 1% increase in PDD_20,10_ ratio, implying a positive correlation between the two factors (*p* < 0.002).

**Conclusions:**

A positive correlation between nominal 10 mm FW and PDD_20,10_ was observed. In the case that the PDD_20,10_ marginally passes the QA tests, users are recommended to consider further verification on Dynamic Jaws to ensure the smallest field width to be within tolerance, which is essential to maintain effective treatment in TomoEDGE system. Since the regression of this study was a single‐factor model, other confounding factors such as the focal spot size of linear accelerator should also be considered when evaluating the machine status.

## 1 INTRODUCTION

In TomoTherapy system, a linear accelerator is mounted on a circular gantry. Treatment is delivered in a helical mode comprising the rotating radiation beam along with concurrent couch movement. A fan‐beam geometry is shaped by adjustable jaws which are further modulated (inplane) by a binary multileaf collimator. The radiation field width is defined by the jaws in the longitudinal (IEC‐y) direction. In TomoTherapy machines without TomoEDGE option, treatment was delivered by static jaws at fixed field width, either 10, 25, or 50 mm at isocenter during the entire treatment. Thus, the field penumbra along the longitudinal direction was approximately identical to the field size on both cranial‐caudal sides of the target volume.^1^ Further to this beam characteristic, it is essential to limit the dose to organ at risk (OAR) by delivering the treatment with MLC collimated sub‐fields under the drawback of increasing treatment time.

**FIGURE 1:**
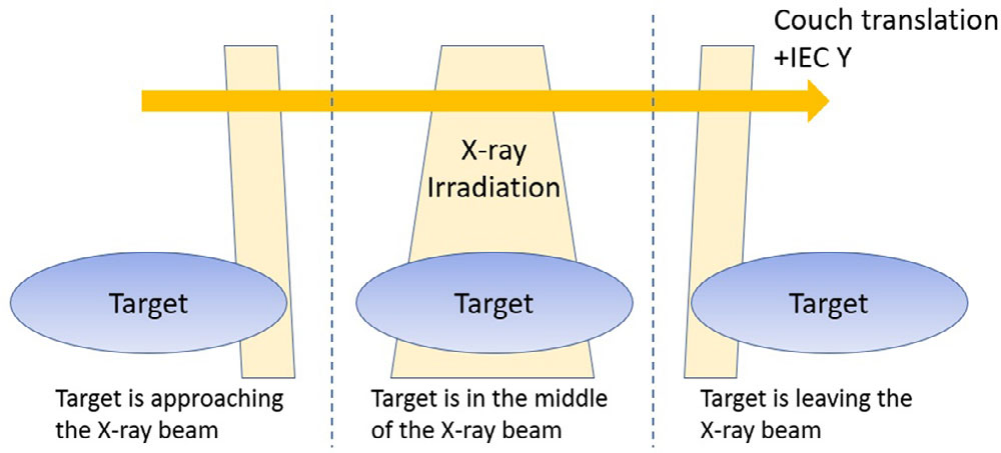
Dynamic jaws illustration.

TomoEDGE is an advanced technology for treatment delivery by introducing a sliding‐window dynamic jaw motion.^2^ The front and back jaws, defining the beam width on the negative IEC‐y and positive IEC‐y direction respectively, move independently at the start and end of a target volume along the longitudinal couch direction. Figure 1 illustrates the concept of sliding‐window dynamic jaw motion. At the start of treatment, the jaws are set close to the minimum width to move across the cranial border of the target. As the target continues to move into the radiation beam, the back jaw keeps opening until the full treatment field size at the center portion of the target is being treated. Then, the front jaws start closing sequentially and reach the minimum jaw width at the caudal border of the target. The treatment delivery is said to be finished when the target volume leaves the radiation beam completely.

With the introduction of dynamic jaw feature, the dose distribution within the target is identical to that with fixed field width but the dose penumbra at cranial and caudal ends of the target is close to the 10 mm field width, irrespective of field size selection. It serves the purpose of reducing the extra dose on patients.

TomoEDGE beam model consists of four symmetric and six asymmetric longitudinal profiles. Full width at half maximum (FWHM) of the beam profile is an important parameter directly related to the accuracy and reproducibility of the dose delivery during tomotherapy treatment.^3^ For the standard governing the quality assurance of helical tomotherapy, AAPM task group 306 (TG‐306) states the importance of measured FWHMs and recommends regular monitoring of the beam parameter to guarantee the agreement with the beam model.^4^

A previous study by Katayama et al. compared the performance between standard static mode and TomoEDGE technique in terms of beam‐on time, average dose on target and dose conformity.^5^ Urso et al. attempted to evaluate the performance in terms of accuracy and precision of the dynamic jaws by measuring all 10 commissioned beam profiles of TomoEDGE technology for a 2‐year duration.^6^ Binny et al. have demonstrated the correlation between the output or energy variations on different delivery modes and patient delivery QA results^7^ while Ferris et al. investigated the effects of variable‐width jaw motion on the beam characteristics for Radixact Synchrony®.^8^ Choi et al. have presented the correlation between TomoTherapy Quality Assurance (TQA) data trend and TomoHD functional status, showing that the exit detector flatness among all four TQA modules (Basic Dosimetry, System Monitor, Step‐Wedge Static and Step‐Wedge Helical) were the most susceptible to beam energy fluctuation.^9^ Until now, no published studies on the evaluation concerning the relationship between TomoEDGE dynamic jaws field width and the energy variations.

This study aims to firstly analyze the performance of the dynamic jaws of all 10 commissioned field widths on an approximately monthly basis on three Radixact tomotherapy units licensed with TomoEDGE for a 2‐year duration. Dynamic jaw performance is defined by the quantitative comparison of FWHM between the golden standard reference and the field width measured in the present study. Secondly, ratio of percentage depth dose (PDD) measured at 20 and 10 cm depth along the static beam central axis in SolidWater are obtained within the same study period. The correlation between the dynamic jaw field width and energy variation is statistically evaluated.

## 2 METHOD

### 2.1 Measurement of dynamic jaws field width

**FIGURE 2:**
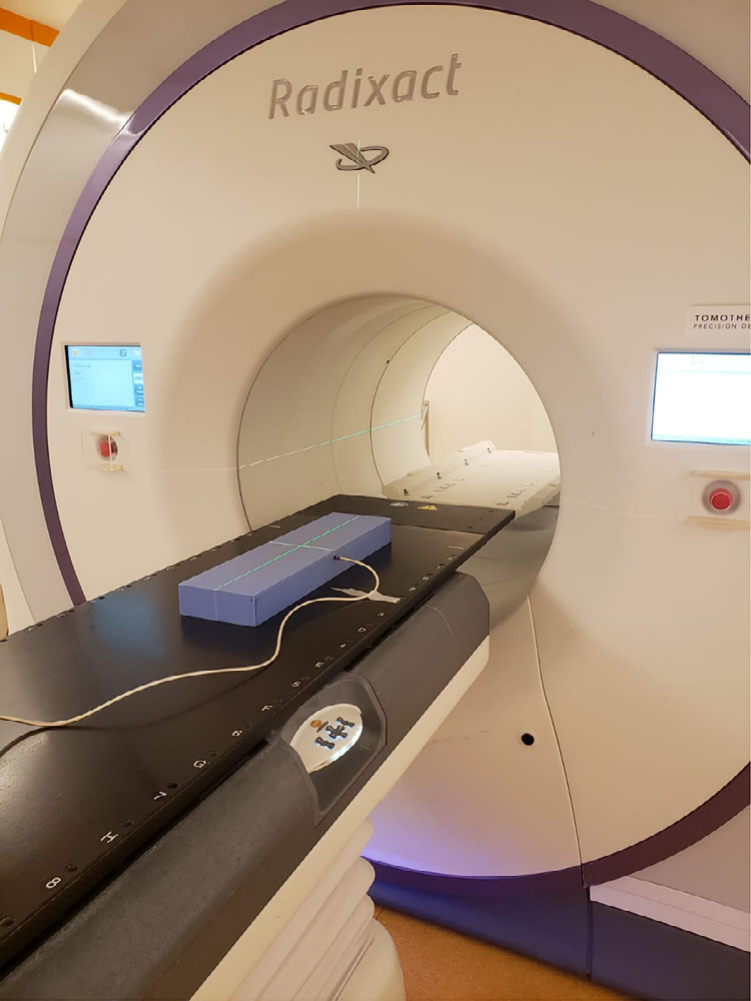
Illustration of the phantom and ion chamber setup.

In this study, three Radixact tomotherapy machines licensed with TomoEDGE were used and Tomotherapy Quality Assurance (TQA) Field Width Dynamic Jaws procedure was employed. It is a standard procedure which performs open‐field measurement of all 10 commissioned longitudinal field width beam profiles sequentially and automatically. In order to measure the topographic field width procedure, an Exradin A1SL ion chamber was inserted into a 5 cm virtual solid water slab phantom. Another 0.5 cm SolidWater slab phantom was positioned on top of the slab containing the ion chamber to ensure the point of measurement was at a depth of 1.5 cm. The slab phantom was placed at a source‐to‐surface distance (SSD) of 85 cm. The height of the couch tabletop was adjusted until the surface of slab phantom was aligned with the gantry bore laser. The setup is illustrated in Figure 2.

**FIGURE 3:**
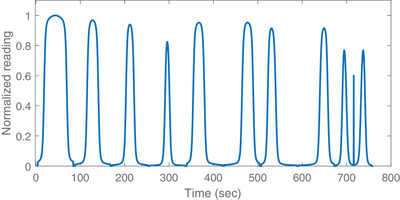
Typical normalized plot of a TQA field width dynamic jaws measurement with the field width order of 50 mm, 25 mm, 20 mm, 10 mm, 25 mm POS, 25 mm NEG, 20 mm POS, 20 mm NEG, 10 mm POS, 10 mm NEG.

To minimize the intrinsic variability in measurement, the same set of ion chambers and SolidWater slab phantom were used throughout the whole study. The beam profiles were collected by the TomoTherapy ElectroMeter Measurement System (TEMS) software by sampling the electrometer reading at regular time intervals when the couch travelled with a constant velocity to move the ion chamber across the radiation beam along the longitudinal direction. Figure 3 shows the typical plot of a TQA Field Width Dynamic Jaws measurement.

For the asymmetric field width, POS notation defined the front jaw and back jaw to be both set on the positive IEC‐y direction with respect to the radiation isocenter while the jaws for NEG were both set on the negative IEC‐y direction.

**FIGURE 4:**
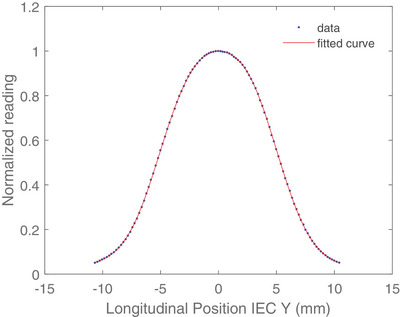
Example of 10 mm field width profile with normalization.

After the acquisition of beam profile data, MATLAB (MathWorks, Natick, MA) was used as the analyzing tool for those reference profiles. Different combinations of Gaussian model were used to fit the profile data and FWHM of each profile was calculated. Figure 4 demonstrates the example of the fitted curve with a field width of 10 mm.

The FWHM results between measured and reference profile were then compared. As recommended by AAPM Task Group 306, the difference between profile's FWHM for symmetric field should not be differed by more than 1% while using an absolute tolerance of 0.5 mm for asymmetric field widths.^4^

### 2.2 Measurement of energy variations

Percentage depth dose (PDD) is an indicator for assessing the beam quality in radiotherapy megavoltage photon beams. On a weekly basis, the PDD ratio of 20 to 10 cm is measured with two Exradin A1SL ion chambers in SolidWater slab phantoms with static beam mode in order to monitor any energy variation of the machines. One of the ion chambers is regarded as a reference chamber to compensate output fluctuations while another is used as the field chamber to measure dose at different depths.

### 2.3 Energy variations study

In this study, the PDD ratio was then used as a metric for the beam energy variation with which measured dynamic jaw field widths were compared. The field widths were plotted against the PDD ratios and were fitted linearly. Pearson's correlation coefficient (denoted as |r|) and the *p*‐values for testing the hypothesis that there was no relationship between the observed phenomena (null hypothesis) were evaluated. A correlation on dynamic jaw field width variation FW can be found based on a two‐year retrospective analysis as below,

(1) $$FW\;=\beta_{1}\;\cdot PDD_{20,10}+\beta_{0}$$

where $PDD_{20,10}$ is the energy variation obtained using an ionization chamber and virtual solid water phantoms at depths 20 and 10 cm, $\beta_{1}$ is the regression coefficient and $\beta_{0}$ is the constant. This equation can thus help to obtain a quantitative study on how the energy variation affects the actual field width in all 10 commissioned field widths.

## 3 RESULT

### 3.1 Verification on the accuracy of dynamic jaws field width

The result of the FWHM comparison for three Radixact tomotherapy units was summarized in Table 1. All measured symmetric field widths in each unit demonstrated to be within 1% tolerance while the absolute differences of all measured asymmetric field widths were within 0.5 mm tolerance. The absolute differences between reference and measured FWs in Radixact A and C are on average 0.06 mm while that in Radixact B is around 0.09 mm.

**TABLE 1:** Field Width FWHM comparison for Radixact Units throughout a 2‐year duration.

		Reference FW	Measured mean ± SD (% Difference)		
			Radixact A	Radixact B	Radixact C
Symmetric FWs	10	10.4583	10.4857 ± 0.05 (+0.26%)	10.4921 ± 0.08 (+0.32%)	10.4099 ± 0.06 (−0.46%)
	20	18.2408	18.1980 ± 0.03 (−0.23%)	18.2824 ± 0.07 (+0.23%)	18.1716 ± 0.03 (−0.38%)
	25	25.1640	25.1640 ± 0.05 (−0.00%)	25.2746 ± 0.09 (+0.44%)	25.1389 ± 0.05 (−0.10%)
	50	50.8208	50.7787 ± 0.07 (−0.08%)	50.9399 ± 0.10 (+0.23%)	50.7710 ± 0.09 (−0.10%)
Asymmetric FWs	10 NEG	9.7822	9.8787 ± 0.05 (+0.98%)	9.8280 ± 0.07 (+0.47%)	9.7650 ± 0.06 (−0.18%)
	10 POS	9.9144	9.8664 ± 0.04 (−0.48%)	9.9720 ± 0.05 (+0.58%)	9.8215 ± 0.04 (−0.94%)
	20 NEG	17.8500	17.8875 ± 0.04 (+0.21%)	17.9046 ± 0.10 (+0.31%)	17.7479 ± 0.05 (−0.57%)
	20 POS	17.9900	17.8353 ± 0.06 (−0.86%)	18.0939 ± 0.08 (+0.57%)	17.8804 ± 0.04 (−0.61%)
	25 NEG	25.1277	25.0698 ± 0.05 (−0.23%)	25.1122 ± 0.10 (−0.06%)	24.9631 ± 0.06 (−0.66%)
	25 POS	25.1707	24.9913 ± 0.07 (−0.71%)	25.1656 ± 0.06 (−0.02%)	25.0407 ± 0.05 (−0.52%)

*Note*: The comparison between the golden standard reference and the measured FWHM in dynamic jaw motion beam model. All values were shown in mm.

Among the measurements of the four symmetric beam profiles, 10 mm FW demonstrated the largest variation between the reference and measured values in all three Radixact units. Percentage of FWHM measurements exceeding the 1% tolerance level (symmetric) or the 0.5 mm tolerance level (asymmetric) in 10 mm FW were listed Table 2. According to AAPM TG‐306, 10 mm symmetric treatment slice width was the most unstable field width to comply with the 1% FWHM tolerance and the test was sensitive to the setup variation.^4^ The failed measurements were repeated promptly after the setup verification such as the examination of the alignment between the surface of slab phantom and the gantry bore laser and couch speed uniformity.

**TABLE 2:** Percentage of individual FWHM measurement exceeding the 1% tolerance level of symmetric FW or 0.5 mm tolerance level of asymmetric FW recommendation by AAPM TG‐306.

Nominal FW (mm)	Tolerance level	Radixact A	Radixact B	Radixact C
10	1% of FWHM	10%	25.6%	20.5%
10 NEG	0.5 mm	0	0	0
10 POS	0.5 mm	0	0	0

*Note*: 10 mm symmetric treatment slice widths were the most sensitive to the 1% FWHM tolerance and the setup should be verified promptly.

**FIGURE 5:**
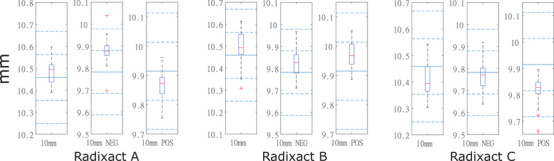
Box plots showing the measured FWHMs (10 mm symmetric and asymmetric) of Radixact Units with the order of Radixact A, B and C. FWHMs were along the vertical axis in mm. On each box, the central mark represented the median, while the top and bottom edges of the box represented the 75^th^ and 25^th^ percentiles, respectively. The outliers were plotted individually using the “+” marker symbol. Standard reference FWHMs were indicated by the central blue solid lines, while dotted and dotted‐dashed lines indicated the ± 1% and ± 2% from standard reference values.

Figure 5 shows the FWHM results of 10 mm symmetric and asymmetric field width in three units graphically. Interquartile range (IQR) is measured by the length of the Tukey boxplot indicating the level of distribution on the measurement. The greater the IQR, the more spread out the measurement. It could be observed that the IQRs of 10 mm symmetric FW were relatively larger in all three Radixact units. Data was tabulated in Table 3.

**TABLE 3:** Statistics of FWHM measurement.

	Radixact A		Radixact B		Radixact C	
Nominal FW (mm)	Median	IQR	Median	IQR	Median	IQR
10	10.4939	0.078	10.4924	0.092	10.3945	0.094
10 NEG	9.8766	0.043	9.8262	0.084	9.7699	0.08
10 POS	9.8731	0.056	9.9673	0.069	9.8276	0.044

*Note*: Interquartile range (IQR) indicated the level of distribution on the measurement.

### 3.2 Energy variation study

In this section, the relationship between the FWHM and energy variation was evaluated. To assess the energy variation, a two‐year retrospective study on the measurement of PDD ratio of 20 to 10 cm depth using SolidWater phantoms was performed. The purpose of the evaluation on PDD_20,10_ ratio was to monitor the energy stability in regular time intervals.

**FIGURE 6:**
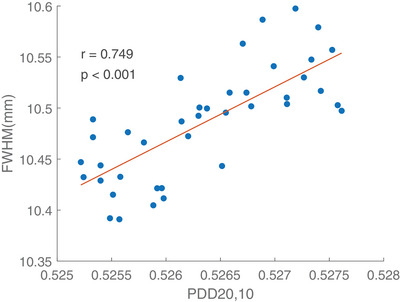
Plot of measured FWHM of nominal 10 mm symmetric FW against PDD_20,10_ for Radixact Unit A.

**FIGURE 7:**
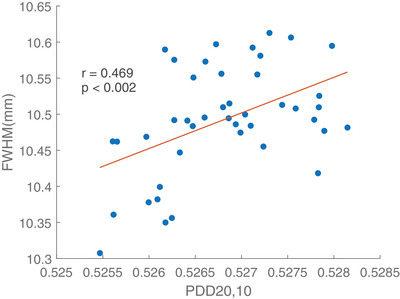
Plot of measured FWHM of nominal 10 mm symmetric FW against PDD_20,10_ for Radixact Unit B.

Linear regression model was fitted to inspect the dependence of energy fluctuation indicated by the PDD ratio of 20 to 10 cm depth on the change of FWHM. Measurements were dependent on several parameters such as the setup, the sensitivity of the ionization chamber and FW. Therefore, same chambers and SolidWater phantoms should be used during the whole study period. The results on the linear regression depicted that an increase in the PDD ratio resulted in a greater measurement on 10 mm symmetric FW, implying a positive correlation between the two factors (*p* < 0.002). Figure 6, 7, 8 shows the energy variation relationship between the measured FWHM of the 10 mm symmetric FW and PDD_20,10_ ratio in Radixact A‐C respectively. The regression coefficients in nominal 10 mm symmetric FW for three Radixact units were summarized in Table 4.

**FIGURE 8:**
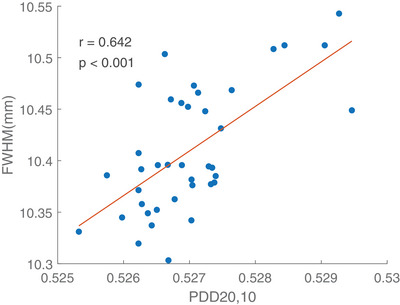
Plot of measured FWHM of nominal 10 mm symmetric FW against PDD_20,10_ for Radixact Unit C.

**TABLE 4:** Regression coefficients and constants in nominal 10 mm symmetric FW.

	Regression coefficient, $\beta_{1}$	Constant $\beta_{0}$
Radixact A	53.955	‐17.9135
Radixact B	49.3523	‐15.5069
Radixact C	43.2831	‐12.4008

The measured FWHM in nominal 10mm symmetric FW showed an increase of 1.5% when PDD_20,10_ ratio was increased by 1%.

## 4 DISCUSSION

### 4.1 Accuracy of dynamic jaws field width

The findings in this study demonstrated the precision and accuracy of the TomoEDGE beam model. Urso et al. have investigated the performance of TomoEDGE technology using a single TomoHDA machine. The results of our study are on the same order with the study presented by Urso et al., where the average absolute difference between reference and measured FWHWs in each Radixact unit was approximately 0.07 mm. It can be observed that around one‐third of the measurement in the small symmetric field width (10 mm nominal FW) did not comply with the 1% tolerance level. As stated by AAPM TG‐306, smaller field width was more sensitive to the external factors of the dynamic jaw movement, for example, the mechanical deviation caused by the noise generated in couch velocity affecting the precision of couch move. Besides, as mentioned by the manufacturer (Accuray Incorporated, Sunnyvale, CA), the dynamic jaw encoder built inherently with an absolute position tolerance of 0.05 mm.^10^ The inherent tolerance was already being one‐half of the recommended 1% tolerance for the field width. As a result, the inherent and external factors contributed to the frequent non‐compliance on the performance of small field width.

### 4.2 Energy variation study

Output energy stability and beam field width are both considered as the important beam parameters in quality assurance procedure of Tomotherapy machine. Binny et al. have evaluated the relationship between the energy variations detected using step‐wedge helical and static modules.^7^ A correlation to patient delivery QA results was demonstrated, suggesting that minor energy fluctuation can affect the dose deliverability. In the aspect of increasing the beam field, the coverage of the total irradiated area increases, and thus extra dose is delivered to the patient which is undesirable. Therefore, monitoring both the energy variation and beam field width are considered as critically important in limiting the extra patient dose.

In this study, the relationship of FWHM and energy variation was carefully investigated. The results depicted that an increase in the PDD ratio resulted in an increase in FWHM on 10 mm symmetric FW, implying a positive correlation between the two factors (p < 0.002).

The evaluation on PDD_20,10_ ratio was used to monitor the energy stability regularly. According to the guideline by the manufacturer (Accuray Incorporated, Sunnyvale, CA), the absorbed dose at depth 20 cm to the maximum absorbed dose at depth 1.5 cm should be in the range 0.315 to 0.327 (0.321 ± 2%) while that at depth 10 cm to 1.5 cm should be in the range of 0.593 to 0.618 (0.6055 ± 2%).^11^ The acceptable criteria of PDD_20,10_ ratio was 0.520 to 0.541 (0.530 ± 2%). It can be demonstrated that even if the ratio of PDD_20,10_ increases but not exceeding the permissible level, it is possible that the beam field width falls outside the 1% tolerance. It is desirable to adjust the range of PDD_20,10_ ratio to verify whether the beam field width pass as well. Based on the linear regression models on three Radixact units, the constrained PDD_20,10_ ratios for Radixact A and B should be 0.524 to 0.528 while that for Radixact C is 0.526 to 0.531. The constraints are machine specified but the range of 0.524 to 0.528 is chosen as the recommended criteria. For the case that the PDD_20,10_ QA marginally passes the constrained range, it is recommended to perform the Dynamic Jaw QA immediately to ensure the smallest field width to be within tolerance, which is essential to maintain effective treatment in TomoEDGE system.

From our study, it can be illustrated that there is a large variation of the measured field width on a small difference of the PDD ratio. The study by Chen et al. revealed that the large variation in measured FWHM for a similar PDD_20,10_ ratio can be the result of multiple factors. The focal spot size of the linear accelerator acts as the major role in affecting the penumbra region. The consequence is especially severe for small fields.^12^ Since the regression of this study was a single‐factor model, other confounding factors such as the focal spot size of linear accelerator should also be considered when evaluating the machine status.

To assess the clinical impact of the variations in beam energy, a circular 2D electronic device was utilized. Nine patient‐specific treatment plans, which carried >90% γ passing rate, were further analyzed. The reference dose was intentionally adjusted by 1% to simulate a slight change in beam energy. γ passing criteria 3% dose/3 mm DTA was set. One out of nine plans reflected a change in passing rate below 90% which would require further investigation in normal clinical settings.

## 5 CONCLUSIONS

Through this long‐term retrospective study, the performance of TomoEDGE technology has been evaluated in three Radixact tomotherapy machines by statistical method. All measured symmetric field widths in each unit demonstrated to be within 1% tolerance while the absolute differences of all measured asymmetric field widths were within 0.5 mm tolerance recommendation stated by AAPM TG‐306. The correlation between FWHM and PDD_20,10_ ratio has been investigated. It is noticeable that an increase in FWHM on nominal 10 mm symmetric FW showed an increase of 1.5% when PDD_20,10_ ratio was increased by 1%. Besides, even the ratio of PDD_20,10_ increases but to be still well within the permissible level, it is possible that the beam field width falls outside the 1% tolerance level. Hence, it is desirable to adjust the range of PDD_20,10_ ratio from 0.524 to 0.528 to verify whether the beam field width also passes as well.

Slight deviation in beam quality QA may be considered as a signal to the change in field width. For the case that the PDD_20,10_ QA marginally passes the constrained range, user is recommended to consider further verification QA on Dynamic Jaw to ensure the smallest field width to be within tolerance, which is essential to maintain effective treatment in TomoEDGE system. Since the regression of this study was a single‐factor model, other confounding factors such as the focal spot size of linear accelerator should also be considered when evaluating the machine status.

## AUTHOR CONTRIBUTIONS

H.M. Poon—Experiment, data analysis, writing—Original draft. T.L. Chiu—Experiment, data analysis, writing—Review & Editing, supervision. S.K. Yu—Conceptualization, resources

## CONFLICT OF INTEREST STATEMENT

The authors declare no conflicts of interest.
